# A world map of esophagus cancer research: a critical accounting

**DOI:** 10.1186/s12967-019-1902-7

**Published:** 2019-05-10

**Authors:** Doris Klingelhöfer, Yun Zhu, Markus Braun, Dörthe Brüggmann, Norman Schöffel, David A. Groneberg

**Affiliations:** 1 Institute of Occupational, Social and Environmental Medicine, Goethe-University, Theodor-Stern-Kai 7, 60590 Frankfurt, Germany; 2 Integrative Medicine Centre, 302 Military Hospital, Beijing, China

**Keywords:** Adenocarcinoma, Cancer epidemiology, Disease burden, Research trends, Funding, Incidence

## Abstract

**Background:**

Esophageal cancer (EC) is one of the deadliest cancers worldwide. The contemporary strong increase of the adenocarcinomas in Western countries and the high mortality rates require the intensification of prospective multinational studies.

**Methods:**

Therefore, this global health issue has been chosen for the bibliometric review of the global publication output. As source for meta and citation data, the Web of Science has been used and Density Equalizing Maps were applied for visualization.

**Results:**

17,387 articles on EC could be identified. The years with publication and citation maxima correspond to the appearance of the most prolific articles. China is the most publishing country, followed by Japan and the USA. Germany and the UK ranked 4th and 5th. The analysis of the ratios articles and socio-economic parameters emphasizes the leading position of the Scandinavian countries and Japan. Here, the high-income countries come out on top. The high incidence regions are mainly represented by Chinese and Japanese research. The association of the publication output and the overall research funding could be shown.

**Conclusions:**

A strengthened international network increasingly consisting of the scientifically best positioned countries as well as more of the high incidence countries worldwide is mandatory for future research. The findings deliver scientists, clinicians and decision makers backgrounds for future decisions all over the world.

## Background

Due to its aggressive behavior and the high mortality rates, esophageal cancer (EC) is one of the deadliest cancers worldwide [1]. With 90%, the main histological types of esophageal cancer are squamous cell- (ESCC) and adeno-carcinomas (EAC) [2]. The treatment and clinical practice of the EC remains a challenge [3, 4] because the importance of histology regarding the decision on the therapeutic approach has still not been settled. In this context, for example the establishment of gene expression profiles must be promote [5].

In 2013, EC ranked 6th as cancer death cause worldwide and regarding the incidence of cancer cases 9th. It is estimated that 455,800 new cases and 400,200 death occurred in 2012 [6]. Men are at higher risk to develop EC than women. The incidence rates depend on the geographical region, sex and race. They vary internationally enormously by more than 21-fold [6] and reach the highest values in Southern and Eastern Africa and Eastern Asia, the lowest values in Western and Middle Africa and Central America [7]. A region called *Esophageal Cancer Belt* reaches from Iran to North-Central China is even since antiquity the highest risk region [1]. In the Western countries (e.g. USA, Australia, UK and France), the rates of EAC has been increasing tremendously, probably due to high prevalence of obesity, Barrett esophagus or chronic GERD (gastroesophageal reflux disease) [8, 9]. In Asia on the other hand, the rate of ESCC increased considerably [8].

The major risk factors are remaining unclear. Even so, smoking, alcohol abuse, deficient or extremely salty diet [10], obesity and Barrett’s esophagus or GERD can be declared as the main risk factors for the development of EC [1, 2]. In some populations, the interaction of poverty or low socioeconomic status [11], nutritionally and social habits seem to increase the risk. Further risk factors of EC are the consumption of very hot liquids [12], the lack of fruits and vegetables [13], the drinking of mate tea through very hot metal tubes, the eating of residues form opium pipes, or chewing of betel nuts [14], to name just a few [1]. The reduced risk by the consumption of acetylsalicylic acid [15] may be explained by the rates of stem cell divisions. Here, the negative effects of acetylsalicylic acid intake must be mentioned as well [16].

The familial accumulation of EC has been shown in high incidence regions such as China [17]. If this is in fact due to a hereditary context is still not resolved until now.

Regarding the estimation of risk factors a differentiation between the ESCC and the EAC is absolutely necessary. Not every risk factor increases the likelihood of getting both types. Alcohol and hot liquids for instance are not linked to EAC, and the reflux diseases do not increase ESCC risk [2].

In contrast to the epidemiologic research on other cancer types [18, 19], there are still many unknown circumstances or uncertainties regarding the epidemiology of EC, e.g. the association between EC and *Helicobacter pylori* is still unclear [20].

The increasing incidence rates all around the world has not reached the zenith yet. The high mortality rate manifests the need to strengthen the research efforts to meliorate the prevention and the treatment of EC. The importance for better monitoring systems has been highlighted in other studies as well [21].

Therefore, the working group took the opportunity to evaluate the research output to depict a new map of the scientific approaches all over the world. The focus has been laid on the international networks, and the development of main topics. In this comprehensive survey the question of what are the most important influences of the research history has been addressed and answered as well as an outlook was given. This is not only important for the scientist but also for the planners, fund raisers and decision makers worldwide.

## Methods

### Methodological platform and data source

This study on the global research output on EC is part of the scientometric platform NewQIS (*New Quality and Quantity Indices in Science*) that has been generated by Groneberg-Kloft in 2009 [22] to provide widespread information on a multitude of biomedical issues.

As data base, the Web of Science of Clarivate Analytics has been used. Its Core Collection supplies the interested and scientific users with publications and the respective bibliometric data. Additionally, it provides the associated citation numbers, so that the analysis of semi-qualitative aspects of the publication output can be carried out. The socio-economic data has been collected from the World Factbook [23].

### Search strategy

The search term has been generated according to the principle of an extensive and representative literature study using the Entry Terms of the MesH Database (*Medical Subject Headings*) of the US National Library of Medicine (National Institutes of Health) [24]. Since these Entry Terms functions as a thesaurus for cataloguing the diseases, the completeness of the search query is guaranteed. The used search term was: “?esophag* AND (*cancer* OR *carcinom* OR *neoplasm*)”. The asterisks replace an indefinite number of characters and has been applied to find different variations of the individual terms. The title search function was used. To include only the original articles in the investigation, the corresponding filter function of WoS was applied.

### Data analysis

The resulting data pool has been integrated in a data base according to its bibliometric information and served as analysis basis. In the focus stood the development of the publication performance regarding EC and its global distribution. The influences over time and the impact of socio-economic features were evaluated too. Additionally, the citation analysis allowed qualitative insights of the scientific efforts and their backdrops. Here, the h-index in a modified version (applied to the performance of countries) and the average citation rate were calculated and analyzed. Usually, the h-index is used as key indicator for the global reputation of an author in the scientific community. It is calculated by the number of publication that at least received the same number of citations each [25]. For further geographical evaluations, the number of articles was put into relation to the gross domestic product (GDP) and the population size of each country. The association between the number of articles and expenditures for research funding, respectively epidemiological conditions set another focus to this study by using linear regression [26, 27]. Furthermore, an analysis of the research areas and author’s keywords has been carried out that permit statements on the main issues, chronologically and geographically.

### Illustration of findings

In part, the findings have been displayed by means of density equalizing map projections (DEMP). This technique represents a method to grab complex global circumstances at one glance by distorting the country sizes. An anamorphic map is the result of the compensation of the osmotic density gradient of the evaluated parameter (e.g. publication number, citation number) [28].

## Results

### Chronological analyses

All in all, the bibliometric data of 17,387 original articles (n) was retrieved. Out of this pool, 16,230 articles were written in English (93.3%). The second most used language was French with 2.47% (n = 430), followed by German (n = 395) and Russian (n = 172).

The number of articles on EC were initially remaining below n = 10 until the end of the Second World War 1945. Afterwards, a moderate but steady increase could be observed until 1980. From then onwards, the numbers increased much faster until they reached their maximum in 2016 with n = 1331 articles. The numbers of citations (c) showed a similar development with peaks in 1994 (c = 10.631) and 2001 (c = 15.258)—until its maximum in 2005 (c = 16.032). Afterwards, the annual amount decreased very rapidly with only small peaks in 2008 (c = 14.951) and 2012 (c = 13.664). Comparing the citation rates (cr) of the publication years, the years 1946 (cr = 56.6), 1998 (cr = 47.5) and 1961 (cr = 41.4) were outstanding.

### Geographical analyses

The information of the country of origin was given in the field tags of 16,686 articles (95.9%). This pool was the source of the geographical analyses and represents the timespan between 1973 and 2017. Previous to this time frame, the geographical data is non-existent, respectively very limited.

**Fig. 1:**
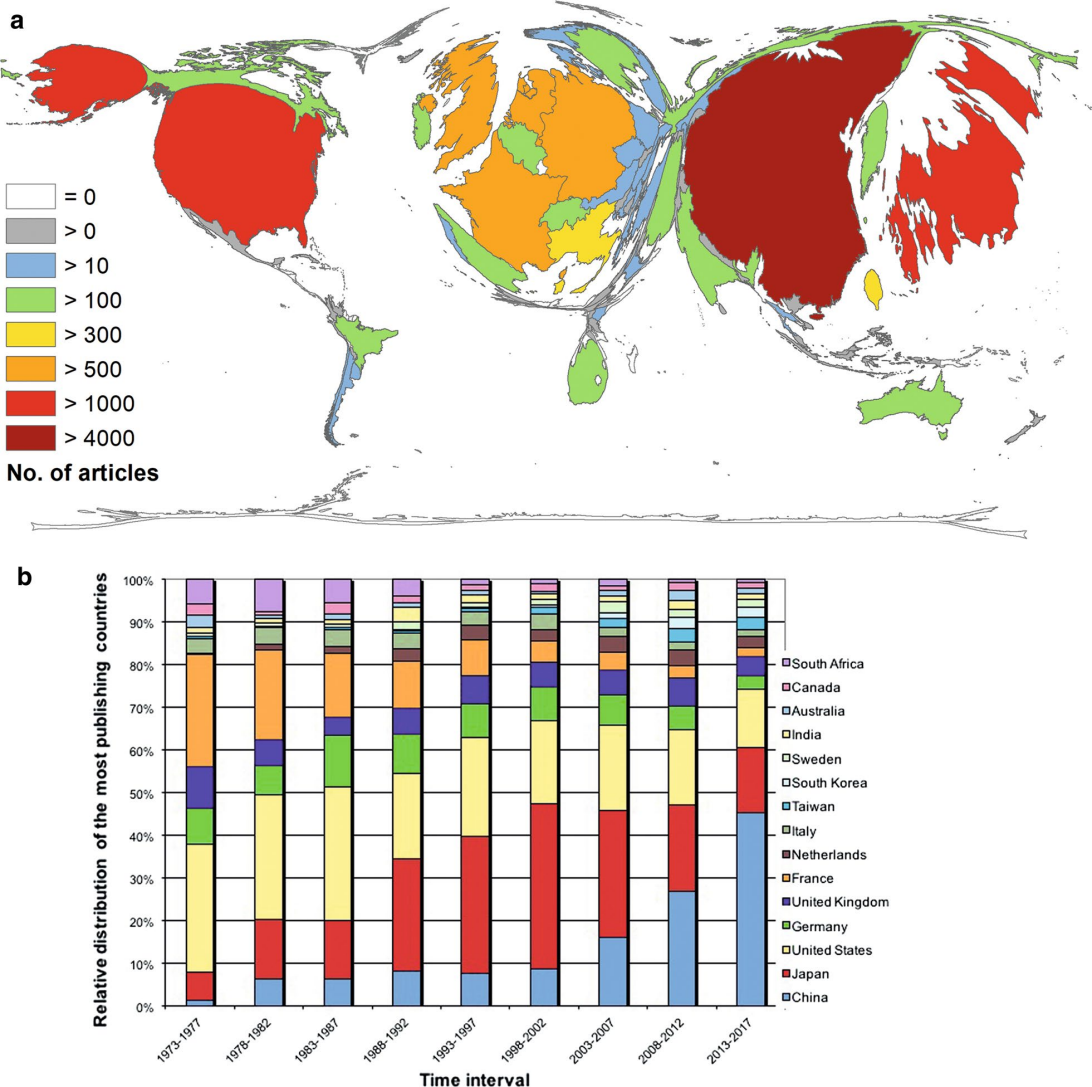
Density equalizing map projection of the absolute numbers of the publishing countries. **a** Number of articles. **b** The development of the relative distribution of the most publishing countries in 5-year intervals from 1973 until 2017

The evaluation of absolute numbers (Fig. 1a) shows that China is the most publishing country with n = 4448 articles on EC, followed by Japan (n = 3828) and the USA (n = 3125). Following behind with some distance, Germany and the UK reached n = 999 and n = 952, respectively.

The chronological development of the relative proportion of the most publishing countries is shown in Fig. 1b. Chinas share increased from 1.39 to 45.30%. In contrast, the USA proportion decreased from 30.09 to 13.53%. The order of the five most publishing countries in the first evaluation interval (1973–1977) is as follows: USA (n = 65), France (n = 57), UK (n = 21), Germany (n = 18), and Japan (n = 14). From 1988 to 2007, Japan published the most part of the OC-articles. The last time evaluation period (2013–2017) is ranked as follows: China (n = 2722), Japan (n = 928), USA (n = 813), UK (n = 268), and Germany (n = 193).

**Fig. 2:**
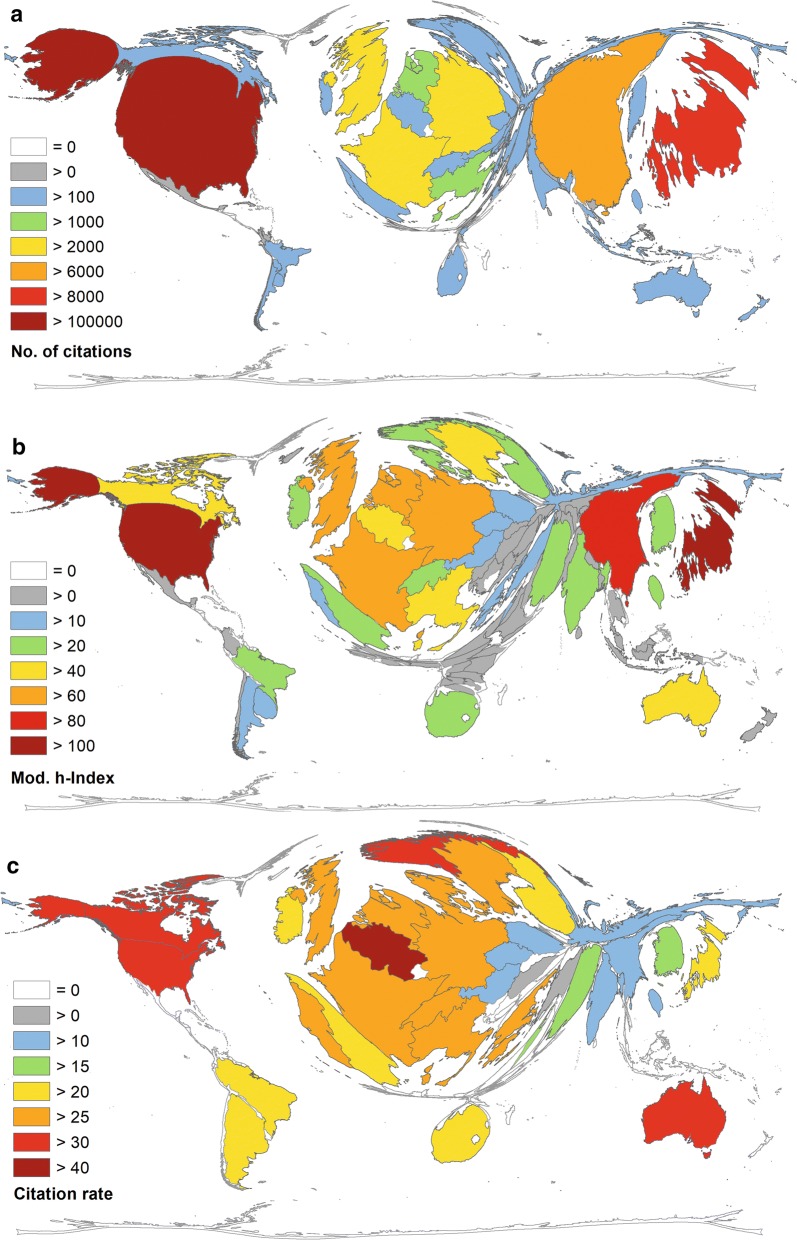
Density equalizing map projection of citation parameters. **a** Number of citations. **b** Modified h-index. **c** Citation rate (threshold ≥ 30 articles)

The USA received the most citations (c = 103,833), followed by Japan (c = 81,099), China (c = 61,726), and also at some distance by the UK (c = 27,607) and Germany (c = 26,095) (Fig. 2a).

Assessing the modified h-indices (hI) of the publishing countries (Fig. 2b) the order of the first five countries is as follows: USA (hI = 134), Japan (hI = 106), China (hI = 89), UK (hI = 78) and Germany (hI = 73). Regarding the average citation rate (cr) of countries with more or equal 30 articles (threshold), Belgium is the leading country with cr = 53.75, followed by Singapore (cr = 38.76), USA (cr = 33.22), Norway (cr = 32.10) and Canada (cr = 31.40) to name the best five (Fig. 2c).

**Fig. 3:**
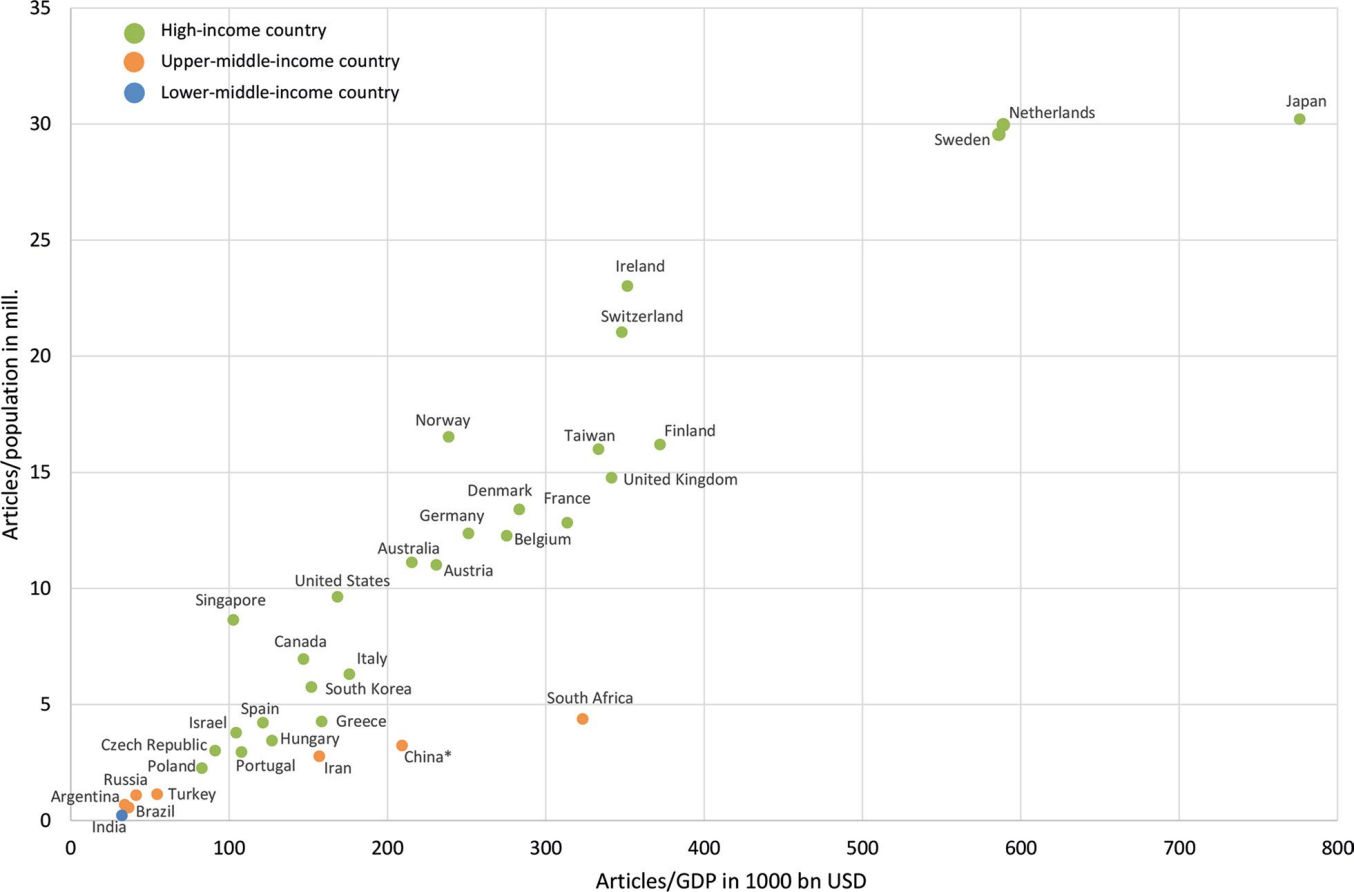
Socio-economic parameters of the countries publishing on esophageal cancer, threshold ≥ 30 articles, *GDP* gross domestic product, *USD* US-Dollar, countries income groups according to the World Bank classification

To give consideration to the differences regarding the number of inhabitants and the economic strength (threshold ≥ 30 articles), the ratios of the number of articles/population in mill. (R_POP_) and the number of articles/GDP in 1000 bn USD (R_GDP_) were analyzed (Fig. 3). It is notable that High-income (HI) countries were ranked higher in principle regarding both parameters, especially Japan (R_POP_ = 30.21, R_GDP_ = 776.15), Netherlands (R_POP_ = 29.97, R_GDP_ = 588.98), and Sweden (R_POP_ = 29.55, R_GDP_ = 586.23). In this analysis, the countries with a lower income level are set back. Especially China—ranking first regarding the absolute evaluation numbers—has been thrown back.

### Influence of research funding and incidence rates

Looking at the association between the publication output of the countries and the overall expenditures on Research and Development in mill. US-Dollars (R&D), [26] respectively the countries’ epidemiological burdens [27] (OECD countries), differences regarding the adequacy of individual research endeavors reveals. For the linear regression of the numbers of articles and the R&D expenditures a coefficient of determination (r^2^) = 0.79 can be calculated. The correlation is strongly significant (Spearman p < 0.0001), while the correlation between the number of articles and the incidence crude rate (ICR) with r^2^ = 0.44 and p = 0.0122 shows only a weak significant association. Therefore, the influence of the funding seems to be stronger that the disease burden or the associated expenses.

**Fig. 4:**
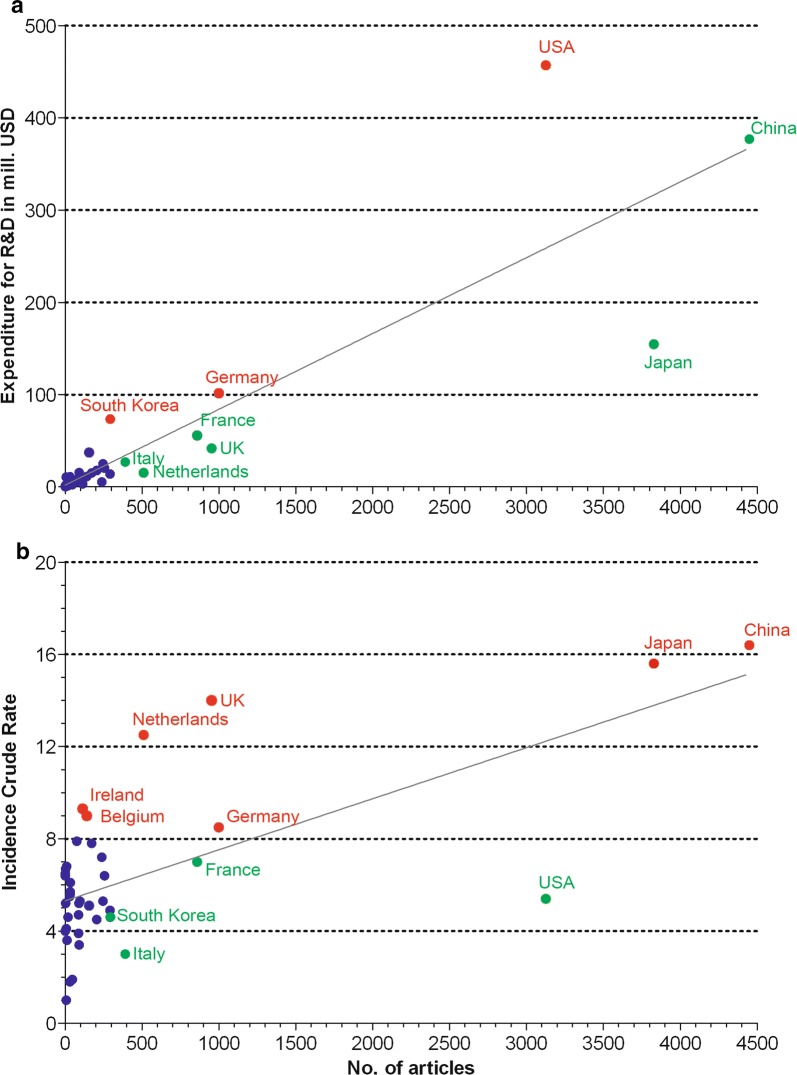
OECD countries. **a** Association between the number of articles and the expenditures for Research and Development (R&D). **b** Association between the number of articles and the incidence crude rate, red spots = negative residuals, green spots = positive residuals, dark blue spots = countries with numbers of articles < 100

**Fig. 5:**
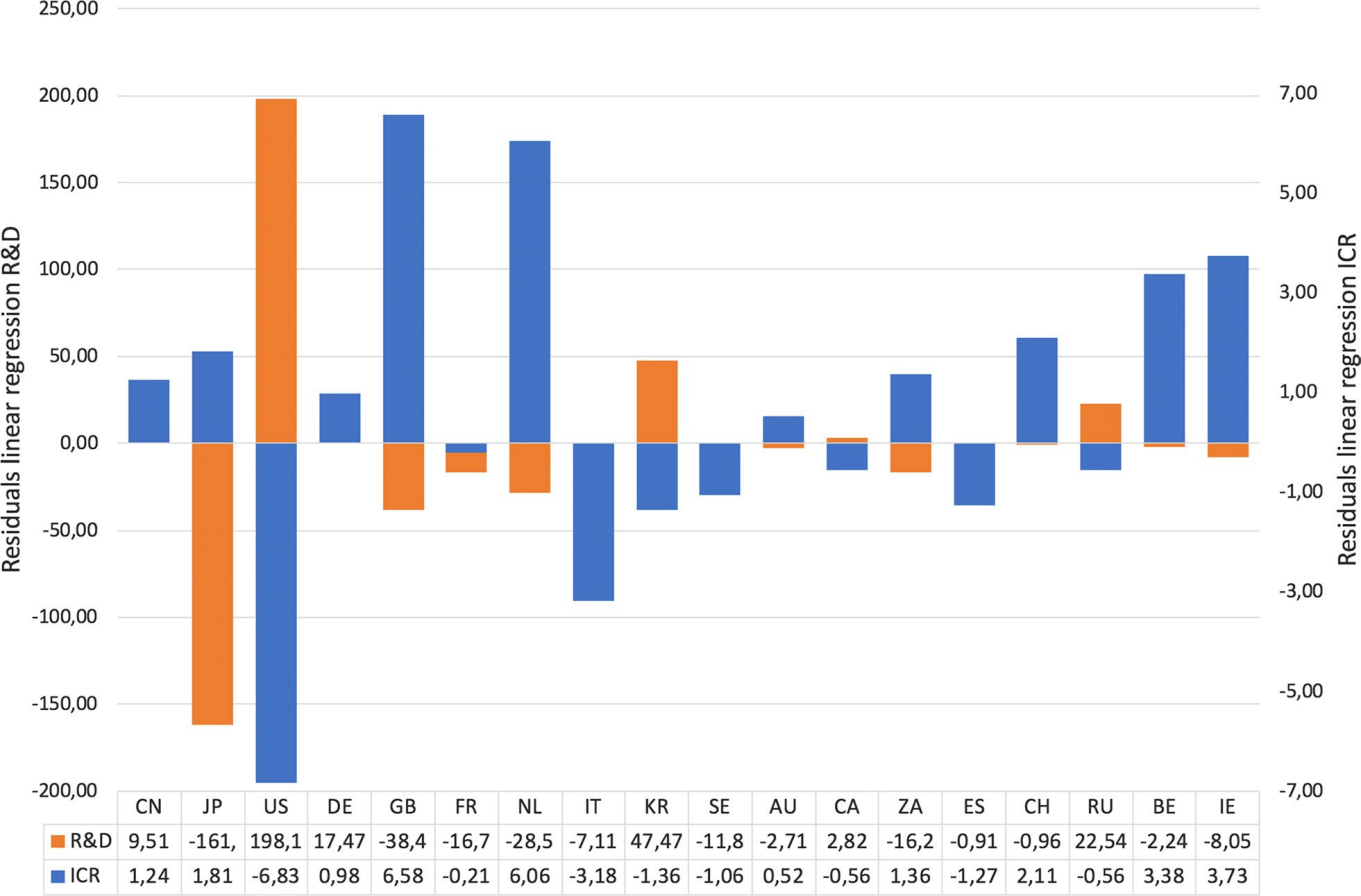
Residuals of the linear regression between the number of articles and the expenditures for research and development in mill. US-Dollar (R&D) and the number of articles and the incidence crude rate (ICR). Threshold = 100 articles. Sorting according to number of articles. Officially countries’ Alpha-2 codes (CN = China, JP = Japan, US = USA, DE = Germany, GB = United Kingdom, FR = France, NL = Netherlands, IT = Italy, KR = South Korea, SE = Sweden, AU = Australia, CA = Canada, ZA = South Africa, ES = Spain, CH = Switzerland, RU = Russia, BE = Belgium, IE = Ireland)

Despite the significant correlation of the funding expenditures and the publication output, the individual countries showed a very different publication behavior (Fig. 4). To analyze the deviations from the regression line, the residuals were calculated and compared (Fig. 5). Striking is the negative position of the USA (Fig. 4a). The published very little compared to their overall research expenditures (R&D = 456.90, n = 3125). In contrast, Japan published relatively much (R&D = 154.71, n = 3828). Additionally, France (R&D = 55.79, n = 858), UK (R&D = 41.78, n = 952), Italy (R&D = 27.05, n = 391) and the Netherlands (R&D = 15.44, n = 510) participated relatively much. China published the most, but with a high funding background (R&D = 376.90, n = 4448). Germany (R&D = 101.58, n = 999) and South Korea showed (R&D = 73.59, n = 293) a slightly negative endeavor on EC research comparing to their overall research expenditure.

Analyzing the publication output regarding the epidemiological EC burden of the OECD countries measured by the ICR, the picture is different (Fig. 4b). Here, UK (ICR = 14) and Netherlands (ICR = 12.5) with a very high rate published only little, while the USA a higher contribution made referring to the values of 2012 [27]. Also, Ireland (ICR = 9.3) and Belgium (ICR = 9) following regarding their ICR were participating very little EC articles. With even higher ICRs, China (ICR = 16.4) and Japan (ICR = 15.6) worked more on EC, although on slightly negative positions.

### Collaboration analyses

**Fig. 6:**
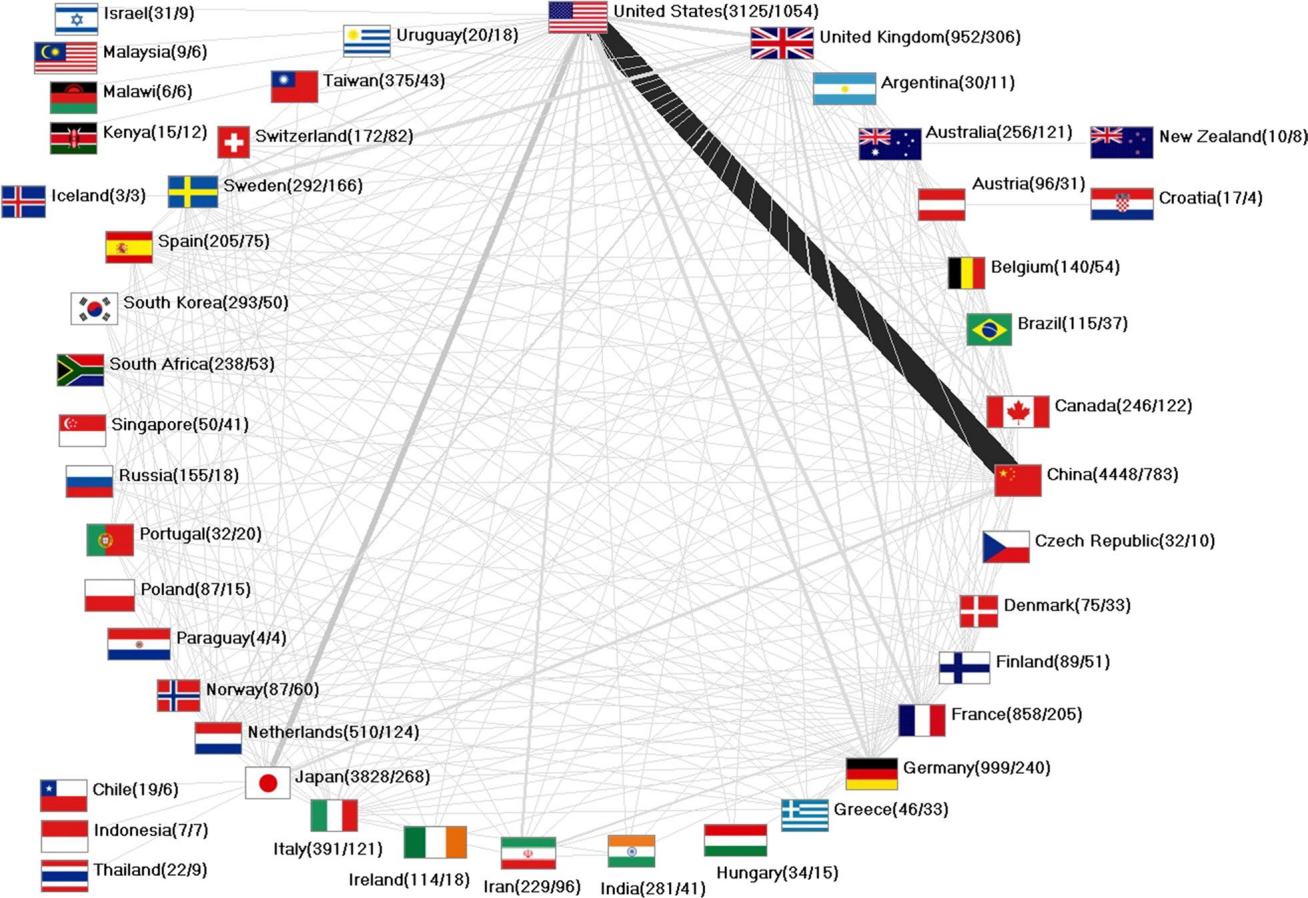
International network of the countries publishing on esophageal cancer, numbers in brackets (number of articles/number of collaboration articles)

The international collaboration network is wide ranging (Fig. 6), but the highest number of cooperation articles were worked out under participation of China and USA with 513 common articles. In addition, the cooperation between Japan and USA with 129 collaboration articles is worth mentioning. China produced 783 of its overall 4448 articles on OC together with another country (17.60%). In contrast, Japan wrote only 7% (n = 268 of 3828) in international cooperation. With 33.73% the USA published more than one-third of its overall articles together with other countries (n = 1054 of 3125), whereof nearly half has been worked out with China (48.87%).

### Research areas

The most assigned subject areas (according to the WoS classification) are *Oncology* with n = 6972 articles, *Surgery* (n = 4282), *Gastroenterology & Hepatology* (n = 2985), *Radiology & Nuclear Medicine & Medical Imagin*g (n = 1126) and *General & Internal Medicine* (n = 1126).

**Fig. 7:**
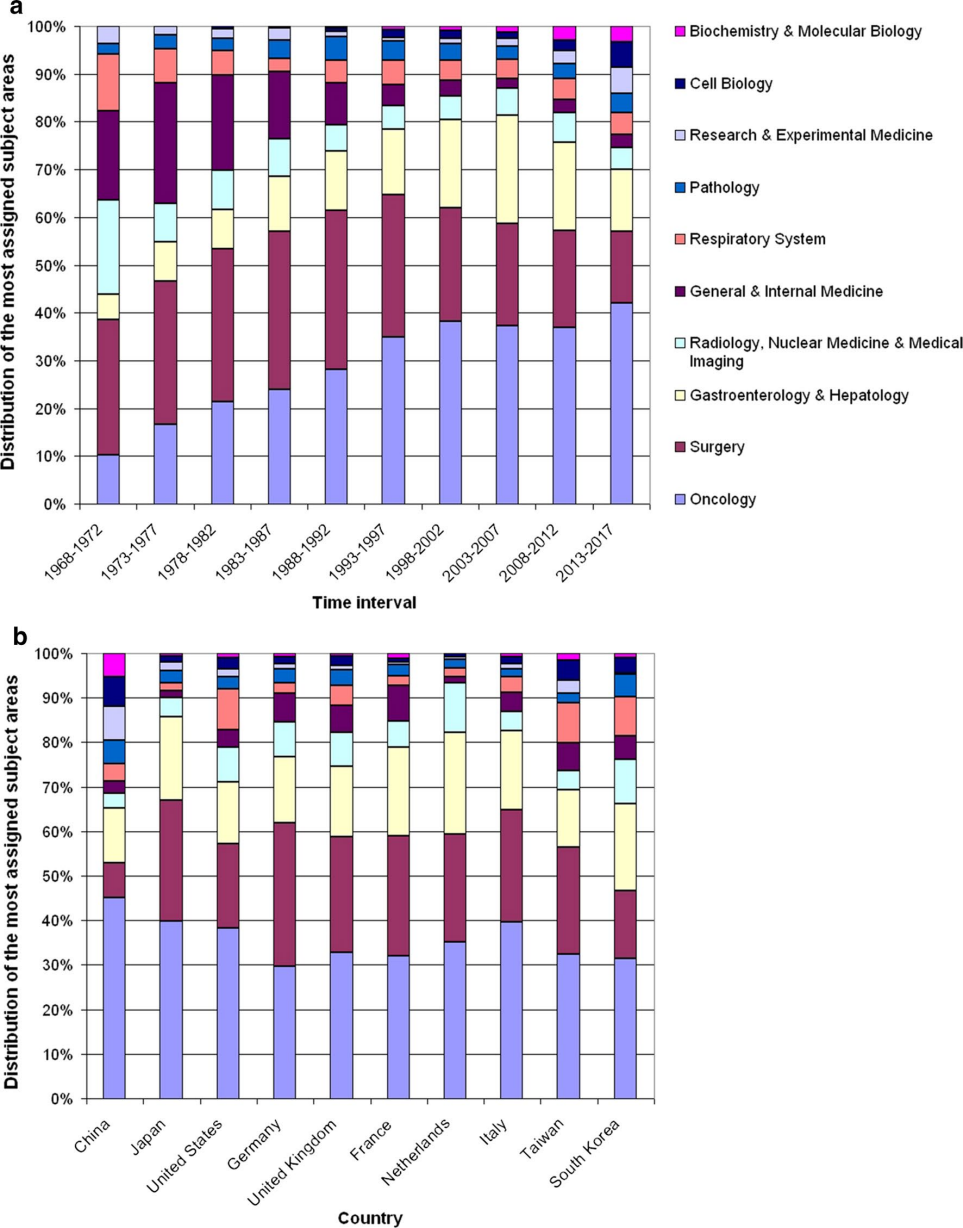
The most assigned subject areas. **a** Relative distribution in 5-year intervals. **b** Relative distribution of the most publishing countries

Looking at the alteration in the relative distribution of the ten most assigned subject areas in 5-year intervals from 1968 until 2017 (Fig. 7a), it can be stated that *Oncology* und *Gastroenterology & Hepatology* relatively increased from 10.16 to 42.13%, while the proportion of *Surgery* decreased from 28.34 to 14.95%. Only since 1993, *Biochemistry & Molecular Biology* appeared among the ten best with only 0.13%, but increased on a moderate level until 2017 (3.21%). The comparison of the most publishing countries (Fig. 7b) revealed differences regarding the proportion of *Surgery* among the ten most assigned subject areas. In China, this area is clearly underrepresented (3.27%), whereas here *Biochemistry & Molecular Biology* is more noticeable than in the other countries.

## Discussion

### Methodical strength and limitations

The enormous amount of scientific publications and the increasing range of journals makes it absolutely necessary to separate the wheat from the chaff. Not only for the individual scientists, but also for planners and funders it is getting more and more difficult to assess the available literature as well as the scientific environment. A long-sighted and globally adapted research is in demand, especially regarding medical issues. So, the NewQIS platform select important medical topics to carry out comprehensive bibliometric reviews. All bibliometric studies depend on the representative status of the database. The WoS is certainly one of the most prolific literature and citation data bases worldwide, but nevertheless, some disadvantages have to be discussed. The English-bias has already been shown in a variety of articles [29, 30]. Even so, possible faulty citing [31, 32] leads to methodological limitation, since the citation numbers are underlying all citation parameters. Therefore, the interplay of the applied citation parameters seems to provide the highest benefit. Likewise, the generation of the applied search term is controversial. The more extensive the data base, the more faultily integrated entries can disturb its quality, and the resulting validity can be brought into question. On the other hand, a data base should be as complete as possible because otherwise it harbours the risk of neglecting important data. Hence, it is a tightrope walk to elaborate a scientifically adequate term and the appropriate strategy.

### Discussion of results

The development of the number of publications follows a known pattern. It has been proofed earlier that the increase of articles number show an exponential progression (Table 1) [33]. Nevertheless, the visible maxima of average citation numbers per year illustrate an association with the most prolific articles (Table 2) [34–37].

**Table 1:** Socio-economic parameters of 10 top ranked countries regarding the R_GDP_ = articles/GDP in 1000 bn UDS (GDP = gross domestic product), R_POP_ = articles/population, total in mill. inhabitants (threshold = 30 articles)

Country	No. of articles	GDP in 1000 bn UDS	Population in mill.	R_GDP_	R_POP_
Japan	3828	4.93	126.70	776.16	30.21
Netherlands	510	0.86	17.02	588.98	29.97
Sweden	292	0.49	9.88	586.23	29.55
Finland	89	0.23	5.50	372.07	16.19
Ireland	114	0.32	4.95	351.53	23.02
Switzerland	172	0.49	8.18	347.97	21.03
United Kingdom	952	2.78	64.43	341.46	14.78
Taiwan	375	1.12	23.46	333.33	15.98
South Africa	238	0.73	54.30	323.24	4.38
France	858	2.73	66.84	313.48	12.84
Denmark	75	0.26	5.59	283.23	13.41
Belgium	140	0.51	11.41	275.27	12.27
Germany	999	3.98	80.72	251.07	12.38
Norway	87	0.36	5.27	238.55	16.52
Austria	96	0.41	8.71	230.82	11.02
Australia	256	1.19	22.99	215.31	11.13
China	4448	21.27	1373.54	209.12	3.24
Italy	391	2.22	62.01	176.05	6.31
USA	3125	18.56	324.00	168.37	9.65
Greece	46	0.29	10.77	158.35	4.27
Iran	229	1.46	82.80	156.96	2.77
South Korea	293	1.93	50.92	151.89	5.75
Canada	246	1.67	35.36	146.95	6.96
Hungary	34	0.27	9.87	127.06	3.44
Spain	205	1.69	48.56	121.30	4.22
Portugal	32	0.30	10.83	107.71	2.95
Israel	31	0.30	8.17	104.38	3.79
Singapore	50	0.49	5.78	102.69	8.65
Czech Republic	32	0.35	10.64	91.19	3.01
Poland	87	1.05	38.52	82.70	2.26
Turkey	91	1.67	80.27	54.49	1.13
Russia	155	3.74	142.36	41.39	1.09
Brazil	115	3.13	205.82	36.68	0.56
Argentina	30	0.88	43.89	34.11	0.68
India	281	8.72	1266.88	32.22	0.22

**Table 2:** The most cited articles

Country	Authors	Years	Citations	Title
USA	Devesa et al.	1998	1484	Changing patterns in the incidence of esophageal and gastric carcinoma in the United States
South Korea, Belgium, Switzerland, China, Japan, Germany, Italy, Russia, Australia	Bang et al.	2010	1312	Trastuzumab in combination with chemotherapy versus chemotherapy alone for treatment of HER2-positive advanced gastric or gastro-oesophageal junction cancer (ToGA): a phase 3, open-label, randomised controlled trial
USA	Herskovic et al.	1992	1301	Combined chemotherapy and radiotherapy compared with radiotherapie alone in patients with cancer of the esophagus
Netherlands	van Hagen et al.	2012	1117	Preoperative chemoradiotherapy for esophageal or junctional cancer
UK	Cunningham et al.	2008	1025	Capecitabine and oxaliplatin for advanced esophagogastric cancer
France, Belgium	Bosset et al.	1997	898	Chemoradiotherapy followed by surgery compared with surgery alone in squamous-cell cancer of the esophagus
USA	Cooper et al.	1999	872	Chemoradiotherapy of locally advanced esophageal cancer—long-term follow-up of a prospective randomized trial (RTOG 85-01)
France	Mandard et al.	1994	842	Pathological assessment of tumor-regression after preoperative chemoradiotherapy of esophageal-carcinoma
USA, Canada	Kelsen et al.	1998	837	Chemotherapy followed by surgery compared with surgery alone for localized esophageal cancer
USA	Urba et al.	2001	813	Randomized trial of preoperative chemoradiation versus surgery alone in patients with locoregional esophageal carcinoma

Additionally, the incidence of EAC in the Western countries has increased tremendously in the last decades [8], so that the significant increase of publications on EC in the last years seems reasonable [38]. The high incidence and prevalence rates of the so called *Esophageal Cancer Belt* leading from Iran, Central Asia to North China cause the high participation of China ranking first. Nearly half of the new OC cases worldwide occurred in China in 2012 [6]. Here, OC ranked 4th regarding the incidence rate after lung, stomach and liver cancer [38] and also regarding the mortality rate [39]. After all, Iran still ranks 15th with 229 articles. Other *belt*-*countries* can be neglected.

The publication output regarding the ratios of the socio-economic factors mat the findings of Man et al. which emphasize the dominancy e.g. of Scandinavian countries, certainly because of the National Cancer Registries that are delivering the clinical data [40–42]. The main difference of both study results is the position of Japan. Despite the low incidence and mortality rate in Japan [43] the research effort on EC is remarkable. Man et al. [44] positioned Japan in the lower part of the ranking regarding the R_POP_ value. In our review, Japan ranks second regarding the absolute numbers of the publication output, and ranks first regarding both socio-economic features (R_POP_, R_GDP_) (Table 2). Regarding the influence of the Total Research Spending, Man et al. [44] found Japan equally high ranked as in our study. This was confirmed by our findings that show that Japan position regarding their expenditures on R&D was extremely positive. It showed a slightly discarded position in comparison to their high ICR. Japan already established several institutions, working groups, and studies with the focus on EC. The Japan Esophageal Society for example, publishing the Esophagus Journal, generated the *Comprehensive registry of EC*. Additionally, the Japan Esophageal Cancer Group has been created as one of the first two Groups of the Japan Clinical Oncology Group (JCOG) in 1978. However, with only 7% international collaborations, for Japan can be calculated the lowest cooperation percentage of all HI-countries publishing on EC.

Despite their ranking among the best five, the publication performance on EC of the USA and the UK is relatively low compared with other studies [45–47]. An analysis of research funding on cancer burden measured by estimated medical costs and years life lost (YLL) stated the underfunding of EC research in both countries [48]. The correlation analysis of this study regarding the connection of the article numbers and the ICR showed a positive participation of US-American scientists based on the Globocan numbers of 2012 [27]. However, there has recently been an extreme accumulation of EAC cases, so that these findings have to be adapted in future. Despite the currently alarming figures of EAC in the US, the NCI decreased the funding of EC research by 15% in 2012. Only about 0.5% of the overall budget was invested. And the American Cancer Society funded only eight OC projects out of 1165 cancer projects [48]. This was confirmed by our findings. They positioned the number of articles on EC in comparison to their overall R&D expenditures last among the OECD countries.

After the findings of this study, the UK positioned itself with a high ICR of 14 and n = 952 article behind. However, the UK works on EC seems to be in line when looking at their overall R&D expenditures. Also, Carter et al. [49] found that the under-funded research on EC, shifted somewhat towards an improved funding from 2000 to 2010. This may be due to the largely nationalized medical system, while in the US only a scarce state health system is established. This led to more prevention measures, more check-ups and more available medical data in UK [49].

Comparing the publication output of other European Countries, a back lying can be stated when looking at the high ICR in 2012. Here, the Netherland, Ireland, Belgium has to be highlighted. Especially the most affected countries in Europa are publishing comparatively little on EC. Looking at the highly affected countries worldwide with ICR over 15, only China and Japan show a justified research endeavor on EC. Other highly affected countries—especially developing countries—did not play a role in the research landscape of EC.

Therefore, the targeted promotion of established science systems and their scientists under the current conditions is highly required and the most affected countries without financial recourses for adequate research efforts have to be supported and included in the global scientific network. Here, a rethink must take place, which leads to a redistribution of resources.

## Conclusions

The unclear epidemiological correlations and the involved bad basis for evaluation complicates the possibility to develop successful prevention measures for EC. Additionally, the extremely geographically and histologically varying incidence rates make the generation of solution approaches very difficult. Therefore, the need for further research seems essential. The increase of new cases in Western countries and the under-funding of EC research shows a significant demand for decision makers, funders and scientists. The low collaboration preparedness of the most acting countries and the weak participation of highly affected countries elucidate the support of countries with an elaborated scientific basis. The challenge in this respect is to optimize research and research funding in accordance with the cancer burden. The establishing of functional health systems can improve the access to reliable data so that adequate strategies can be developed. There is a large demand for multidisciplinary approaches to fulfil the complex scientific issues related to EC.
